# Historical Temperature Variability Affects Coral Response to Heat Stress

**DOI:** 10.1371/journal.pone.0034418

**Published:** 2012-03-30

**Authors:** Jessica Carilli, Simon D. Donner, Aaron C. Hartmann

**Affiliations:** 1 Institute for Environmental Research, Australian Nuclear Science and Technology Organization, Lucas Heights, New South Wales, Australia; 2 Department of Geography, University of British Columbia, Vancouver, British Columbia, Canada; 3 Scripps Institution of Oceanography, University of California San Diego, La Jolla, California, United States of America; King Abdullah University of Science and Technology, Saudi Arabia

## Abstract

Coral bleaching is the breakdown of symbiosis between coral animal hosts and their dinoflagellate algae symbionts in response to environmental stress. On large spatial scales, heat stress is the most common factor causing bleaching, which is predicted to increase in frequency and severity as the climate warms. There is evidence that the temperature threshold at which bleaching occurs varies with local environmental conditions and background climate conditions. We investigated the influence of past temperature variability on coral susceptibility to bleaching, using the natural gradient in peak temperature variability in the Gilbert Islands, Republic of Kiribati. The spatial pattern in skeletal growth rates and partial mortality scars found in massive *Porites sp.* across the central and northern islands suggests that corals subject to larger year-to-year fluctuations in maximum ocean temperature were more resistant to a 2004 warm-water event. In addition, a subsequent 2009 warm event had a disproportionately larger impact on those corals from the island with lower historical heat stress, as indicated by lower concentrations of triacylglycerol, a lipid utilized for energy, as well as thinner tissue in those corals. This study indicates that coral reefs in locations with more frequent warm events may be more resilient to future warming, and protection measures may be more effective in these regions.

## Introduction

Coral bleaching is a stress response in which corals lose their symbiotic dinoflagellate algae [1]. On large spatial scales, bleaching usually occurs when heat stress causes a breakdown in symbiont photosynthesis that leads to the production of oxygen radicals [2], [3]. Early work suggested that coral bleaching may occur when water temperatures exceed the maximum normally experienced in the average year by 1°C for a month or more [4]. The accumulation of temperature stress in excess of coral bleaching thresholds, often expressed as the accumulation of degree-heating-months (DHM) or degree-heating-weeks (DHW), is commonly used to predict mass bleaching events [5], [6]. Observations indicating that mass bleaching events have recently become more common [7]–[12], combined with projected increases in heat stress, have prompted dire predictions for the future of coral reefs under unabated greenhouse gas emissions scenarios [13], [14].

Understanding of the factors that affect coral bleaching thresholds is useful to improve both predictions of bleaching severity due to a given amount of heat stress [15], [16], as well as to investigate whether local short-term mitigation of other stressors or protection of more resistant reefs can delay or avoid degradation of coral reefs over the next several decades [11], [17], [18]. Recent work has shown that bleaching thresholds in individual corals are not simply related to a certain threshold DHW, nor are the bleaching thresholds necessarily static over time. For example, bleaching susceptibility varies between taxa [19]–[21], as well as within coral taxa containing different symbiont types [22], [23]. Other sources of stress such as nutrient runoff, sedimentation, overfishing, and ocean acidification appear to interact with heat stress to change the bleaching threshold [11], [18], [24], [25]. In addition, there is evidence that corals may adapt to better withstand heat stress via a number of mechanisms. Corals might acquire more thermally-resistant symbionts [26], [27], or might increase their own physiological mechanisms to reduce bleaching susceptibility by producing oxidative enzymes [28] or photoprotective compounds [29]. On a reef-wide scale, more resistant taxa may increase in dominance after bleaching [30], [31].

There is also evidence that the susceptibility of a given coral or reef to bleaching depends on the thermal history [15], [16], [32]. Several experimental studies have investigated the influence of prior exposure on the ability of corals to withstand heat stress. One study found that coral nubbins collected from the same colonies on the Great Barrier Reef (GBR) and pre-exposed to slightly elevated temperatures subsequently experienced less zooxanthellae loss than controls when experimentally bleached [33]. Similarly, two studies that collected samples from coral colonies inhabiting environments with different temperature ranges found that corals from more variable environments were less affected by heat stress in the laboratory [34], [35]. Other studies have found that prior thermal stress reduced the impact of subsequent heat stress events on coral communities on Palmyra Atoll in the central Pacific [36], several different reefs on the GBR [37], and worldwide using globally-gridded sea surface temperature (SST) products [15]. However, to date, no field studies have specifically investigated how thermal history affects bleaching susceptibility in individual corals during real-world heat stress events.

**Figure 1:**
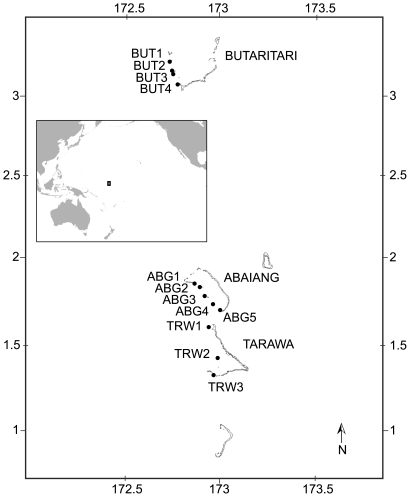
Map of sites studied. The box in the inset of the larger Pacific Ocean shows the location of the site map.

In this study, we collected cores from massive *Porites sp.* corals in the Gilbert Islands of Kiribati to investigate how corals along a natural gradient in temperature variability responded to recent heat stress events. The Gilbert Islands are a group of 15 atolls and reef islands that span the equator from about 3.5°N to 2.5°S in the central Pacific (Figure 1). Unlike much of the tropics, the Gilbert Islands experience high inter-annual variability in peak temperatures due to the effect of the El Niño-Southern Oscillation (ENSO), but low average seasonality in temperatures due to their near-equatorial position [16]. The magnitude of variability in maximum annual temperature, and the frequency and intensity of thermal stress, decreases from the equatorial Tarawa Atoll (1°N) to the more northerly Butaritari Atoll, which is less influenced by the shifts in trade winds and surface currents that occur during ENSO events (3.5°N) [16]. This ENSO-driven latitudinal gradient provides an ideal laboratory for evaluating the influence of past temperature variability on coral susceptibility to bleaching due to heat stress.

We examined changes in coral skeletal growth rates and partial mortality scars [11] to investigate the impact of the bleaching event in 2004 [16] on corals from different temperature variability regimes. Skeletal extension rate reductions have been noted in corals due to bleaching in several regions [10], [38], [39]. However, apparently only the more severe bleaching events cause a reduction in skeletal growth rates; for instance, one study found growth anomalies in 95% of corals from the Mesoamerican Reef related to severe bleaching in 1998, though only a single core (of 92 collected) showed an anomaly due to a less severe event in 1995 [11]. We also investigated differences in lipid class composition, namely the concentration of triacylglycerol and wax esters, the primary energetic lipids utilized by corals [40], to investigate the impact of a second heat stress event in 2009. We found that corals historically exposed to lower thermal variability were more severely affected by thermal stress in both 2004 and 2009, indicating that reefs experiencing more naturally variable temperature regimes may be more resistant to future warming.

## Methods

We collected coral cores and tissue samples from several locations around three atolls in the central and northern Gilbert Islands: Tarawa, Abaiang, and Butaritari, with permission and assistance from the Republic of Kiribati's Ministry of Fisheries and Marine Resource Development (Figure 1).

### Coral core collection

A total of 28 cores were collected during May, 2010. The cores were all collected at approximately 5 m depth on the fore-reef (except for the core in the Tarawa lagoon, which was ∼1 m deep at low tide). We used a hand-held reversible air drill driven by a gasoline-powered air compressor. A custom-built stainless steel core barrel 6 cm in diameter and 50 cm long fitted with a brass drill head containing carbide teeth was used, fashioned after a design developed by the Australian Institute for Marine Science. After core removal, pre-cast concrete plugs were inserted to prevent colonization of the inside of the coral by boring organisms and allow coral overgrowth. At each site, we collected cores from the largest heads within reach of a 30-m-long air hose. Cores were drilled vertically to obtain the clearest growth banding pattern along the maximum growth axis. After collection, cores were rinsed in fresh water and air-dried. Cores were imported to Australia under CITES permit #2010-AU-594729.

### Coral tissue sample collection

Small ∼1 cm cubes of coral tissue and skeletal material for lipid analysis were collected from 3–5 coral heads at a site using a hammer and chisel or a metal punch with permission and assistance from the Republic of Kiribati's Ministry of Fisheries and Marine Resource Development. Not all sites where cores were collected were sampled for coral lipids due to logistical constraints. Samples were collected at 5 m depth; at one site on Abaiang, samples were also collected at 10 m depth. Samples were wrapped in aluminum foil and either frozen in a household freezer (Tarawa and Butaritari) or kept on ice before freezing (Abaiang; 2 days on ice) and were subsequently transported on ice to ANSTO, where they were stored in a −20°C lab freezer until processing. Samples were imported to Australia under CITES permit #2010-AU-594729.

### Measurement of coral growth rates and tissue thickness

Cores were scanned whole using computerized tomography (CT) at the Royal North Shore Hospital in Sydney, Australia with a Siemens Biograph mCT [41]. Images were taken in 0.6 mm axial slice increments using a 100 mm field of view, 140 kV and 300 mAs. Images were reconstructed at the hospital using a “bone” window and ultra-sharp reconstruction and exported as DICOM files for processing in the laboratory.

**Figure 2:**
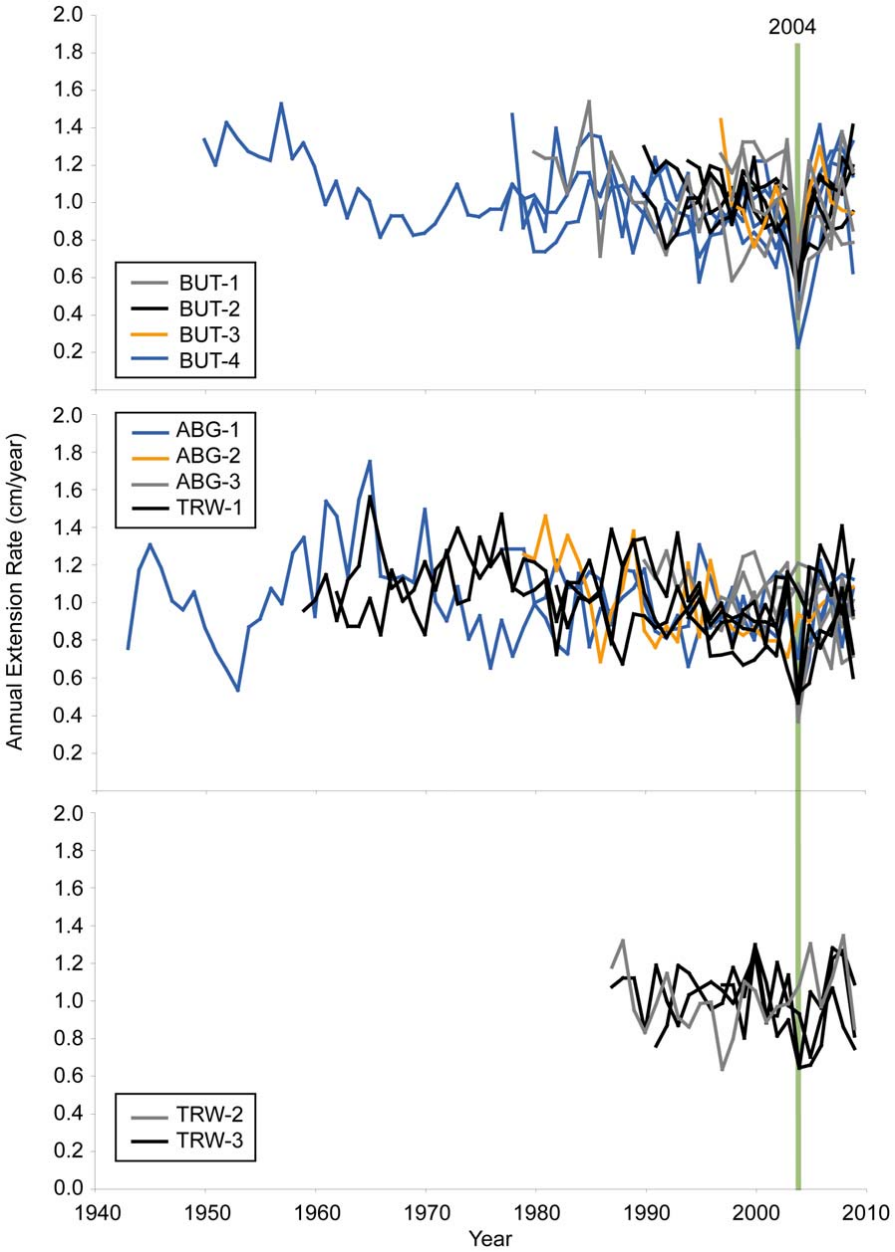
Annual coral extension rates. Extension rates for individual coral cores standardized such that the long-term average for each record is equal to 1 cm/year.

We used the open-source program Osirix (version 3.8.1 with 64-bit extension) to reconstruct 3-d images of core density from CT scan data using the maximum intensity projection mode. We then selected the maximum growth axis and took a virtual 3.4-mm thick slice through the core along this axis, revealing the annual density banding in each core. We used the “length” tool in Osirix to select and extract density data in Hounsfield units on transects perpendicular to the clearest growth banding. Hounsfield units were converted to density using CT scans of aluminum wedges originally designed for calibrating x-ray density (see supplemental material in [11]). Annual bands were then identified manually between density minima, and the annual extension (cm/year), density (g/cm^3^/year), and calcification (extension * density; g/cm^2^/year) rates were calculated for the core. This was done twice along the length of each core in different locations, averaging the two series to construct the final growth record for each core, and finally standardizing to an average extension rate of 1 cm/year by dividing by the mean for each series (Figure 2). See Table S1 for average data before standardization from each site. Extension rates are presented here (Figure 2), as changes in calcification rates are mainly driven by extension due to minimal density fluctuations in these cores (Figure S1, S2); this has been found in other studies as well [11], [42]. Partial mortality scars were recognized by comparing anomalous features such as truncated density bands and very dense material in the CT scans with the original cores [43]. Tissue thickness was measured as the depth in the skeleton occupied by tissue, recognized visually, using calipers on the original cores [44].

### Coral total lipid extractions

Coral lipids were extracted using a modified Folch procedure [45]. Coral samples were first ground in a mortar and pestle, then lipids were allowed to extract in the dark for one hour in a 2∶1 (v/v) chloroform∶methanol solution. Samples were then filtered and separated from remaining non-lipid material in a separatory funnel by sequential addition of 0.88% KCl and 100% chloroform. Finally, samples were dried under nitrogen and weighed to determine the dry lipid mass. Filters were dried, weighed, combusted at 450°C for 6 hours, and reweighed to determine ash-free dry weight of non-lipid fraction. Total lipid ratios were calculated as the mass of dry lipids/biomass of the coral (which consisted of the lipid mass plus the ash-free dry weight).

After drying, each sample was re-suspended in 750 µl of chloroform, from which 1 µl was spotted at the origin of each of three quartz Chromarods (S-III, Iatron Laboratories, Inc.). Lipid classes were separated chromatographically by developing the rods in a sequential two solvent system: 1) hexane∶ethyl ether∶acetic acid (99∶1∶0.05) (v∶v∶v) for 25 minutes; 2) hexane∶ethyl ether∶acetic acid (80∶20∶0.1) (v∶v∶v) for 25 minutes, a modification to the methods in [40]. Chromatograms were then generated via flame ionization detection (FID) of the full length of each rod using an Iatroscan TLC-FID MK-5 (Iatron Laboratories, Inc.) and LabView software (National Instruments). The triplicate analyses made for each crude lipid sample were averaged. Lipid class concentrations were calculated by comparing sample peak areas and retention times against previously generated calibration curves and retention times of known standards. The standard compounds used were: 5-α-cholestane for hydrocarbons, palmitic acid palmityl ester for wax esters, tripalmitin for triacylglycerol, stearic acid for free fatty acids, stigmastanol for sterols and L-α-phophatidylcholine for phospholipids.

### Thermal variability and stress

**Figure 3:**
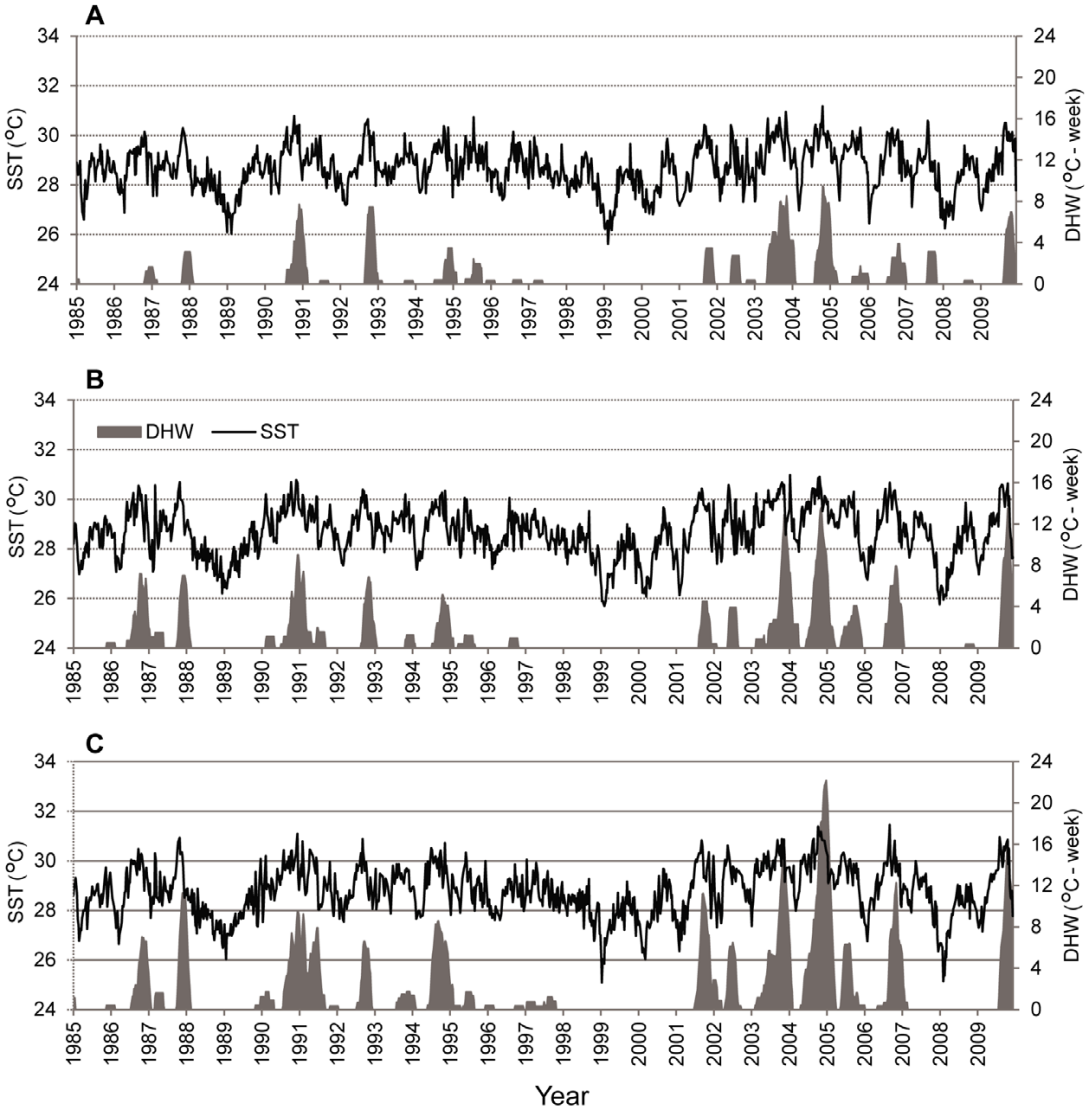
Weekly sea surface temperature (°C; black) and degree heating weeks (°C·week; gray) from 1985 through 2009. Plots show the average of the values from the 4 km^2^ AVHRR grid cells that include field sites in (A) Butaritari, (B) Abaiang and North Tarawa, and (C) South Tarawa.

Weekly sea surface temperature (SST) data at 4-km resolution were obtained from the AVHRR Pathfinder satellite retrospective dataset (1985–2009) made available by the NOAA Coral Reef Watch Program (http://coralreefwatch.noaa.gov; Figure 3). We calculated three metrics of thermal history: (1) the mean of the annual maximum DHW from 1985–2003 (2) the proportion of years from 1985 to 2003 in which the maximum DHW exceeded 4°C·week, and (3) a year-to-year temperature variability metric from [16], [46], which is the standard deviation of the maximum monthly SST from 1985–2000 scaled such that the mean for the world's coral reefs is 1°C. We also calculated heat stress in 2004 as the maximum DHW at each site.

### Statistical analyses

We measured the severity of bleaching in 2004 as (1) the percentage that extension rates were reduced in 2004 from the long-term average for the length of each coral core up to 2003 and (2) the proportion of cores that showed a partial mortality scar in 2004. We used lipid/biomass ratios and tissue thickness as measures of coral health at the time of collection [25], [44]. Because of the similar thermal history and close proximity of Abaiang and North Tarawa, we grouped sites from those islands together to compare against sites at Butaritari. Data from the site adjacent to heavily populated South Tarawa (TRW3) were compared separately to investigate differences that may be associated with the extreme local human impacts at South Tarawa. We tested for normality of the data using the Shapiro-Wilkes test in R (version 1.13). We used t-tests when data were normal and permutation tests when data were not normal to test for differences in bleaching severity, tissue thickness, lipid measures, and skeletal growth rates between corals from the island groups. We also tested for significant differences in thermal variability measures as well as heat stress in 2004 and 2009 between these island groups.

## Results

Background thermal variability from 1985–2003 was lower at Butaritari than at Abaiang and North Tarawa as well as at South Tarawa (Table 1). Using permutation tests between temperature metrics at Butaritari versus Abaiang and North Tarawa, we found significant differences in the mean of the maximum annual DHW (mean 2.3°C·week versus 3.9°C·week, p<0.01) and the scaled year-to-year temperature variability metrics (mean 1.3°C·week versus 1.5°C·week, p<0.01). The mean maximum DHW in 2004 was higher at Abaiang and North Tarawa (mean of all sites 14.6°C·week) than at Butaritari (mean 10.4°C·week). Likewise, though less warming occurred overall in 2009 compared to 2004, the mean maximum DHW in 2009 was significantly higher at Abaiang and North Tarawa (mean of all sites 13.8°C·week) than at Butaritari (mean 7.1°C·week) (p<0.01, t-test). In both 2004 and 2009, South Tarawa experienced the highest heat stress, with maximum DHW of 24.2°C·week and 19.1°C·week, respectively (Table 1).

**Table 1:** Thermal history metrics calculated for each site.

Island	Site	Mean Maximum DHW from 1985–2003	Proportion Years from 1985–2003 with DHW >4	Temperature Variability Metric	Maximum DHW in 2004	Maximum DHW in 2009
Butaritari	BUT1	2.46	0.26	1.56	7.15	5.65
	BUT2	2.34	0.26	1.28	10.84	7.85
	BUT3	2.22	0.21	1.16	11.83	7.30
	BUT4	2.22	0.21	1.16	11.83	7.30
Abaiang	ABG1	3.49	0.26	1.49	9.59	13.52
	ABG2	3.49	0.26	1.49	9.59	13.52
	ABG3	3.40	0.37	1.52	17.06	15.41
North Tarawa	TRW1	3.36	0.42	1.72	15.23	11.63
	TRW2	5.31	0.53	1.71	21.62	14.70
South Tarawa	TRW3	5.17	0.47	1.62	24.16	19.07

DHW stands for degree-heating-weeks, the integral of sea surface temperature (SST) elevated above the maximum climatological mean over time (°C·week). The temperature variability metric is the standard deviation of the maximum monthly SST from 1985–2000 scaled such that the mean for the world's coral reefs is 1°C [16].

**Figure 4:**
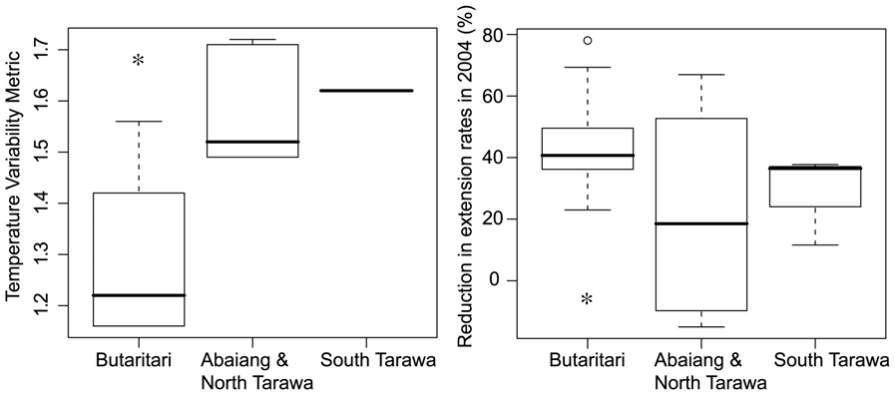
Boxplots of temperature variability and reduction in coral extension rates in 2004. The temperature variability metric at each site was calculated as the standard deviation of the maximum monthly SST from 1985–2003, scaled such that the mean for the world's coral reefs is 1°C [16]. Coral growth rate reductions are presented as the percentage that extension rates were reduced in 2004 compared to all previous years for each individual core. Asterisks indicate significant differences between measures at Butaritari versus Abaiang and North Tarawa.

Corals from Butaritari were more severely affected by bleaching in 2004 (Figure 2); on average corals from Butaritari had a 45% reduction in skeletal extension rates in 2004 compared with a 22% reduction at Abaiang and North Tarawa (p = 0.055, permutation test between sites, Figure 4). Density changes were small and inconsistent (for example, density in 2004 increased compared to previous years by 2% on average at site BUT3, but decreased by 3% on average at BUT9). Calcification (extension * density) changes were therefore almost identical to extension. At Butaritari, all corals had reduced extension in 2004 compared to all previous years for each individual coral, ranging from 22–78%. At Abaiang and North Tarawa, five corals actually had higher extension in 2004 (maximum 15% increase compared to previous years), while the largest reduction was 67% in one core. In addition, 31% of corals from Butaritari (N = 13) had partial mortality scars associated with the 2004 bleaching event, while none from Abaiang and North Tarawa showed such scars (N = 12); this difference was marginally significant (p = 0.10, permutation test). There were no significant differences in the magnitude of extension rate reduction or partial mortality between Abaiang and North Tarawa versus South Tarawa.

Skeletal extension rates recovered within two years of the bleaching event at both island groups (Figure 2). We did not find any evidence for prior bleaching events in our cores; there were neither partial mortality scars prior to 2004 nor any significant reductions in growth rates in multiple cores in any single prior year. The majority of cores were not long (<30 years, Figure 2) and therefore prior events may have occurred but were not recorded in the few longer cores we collected.

**Figure 5:**
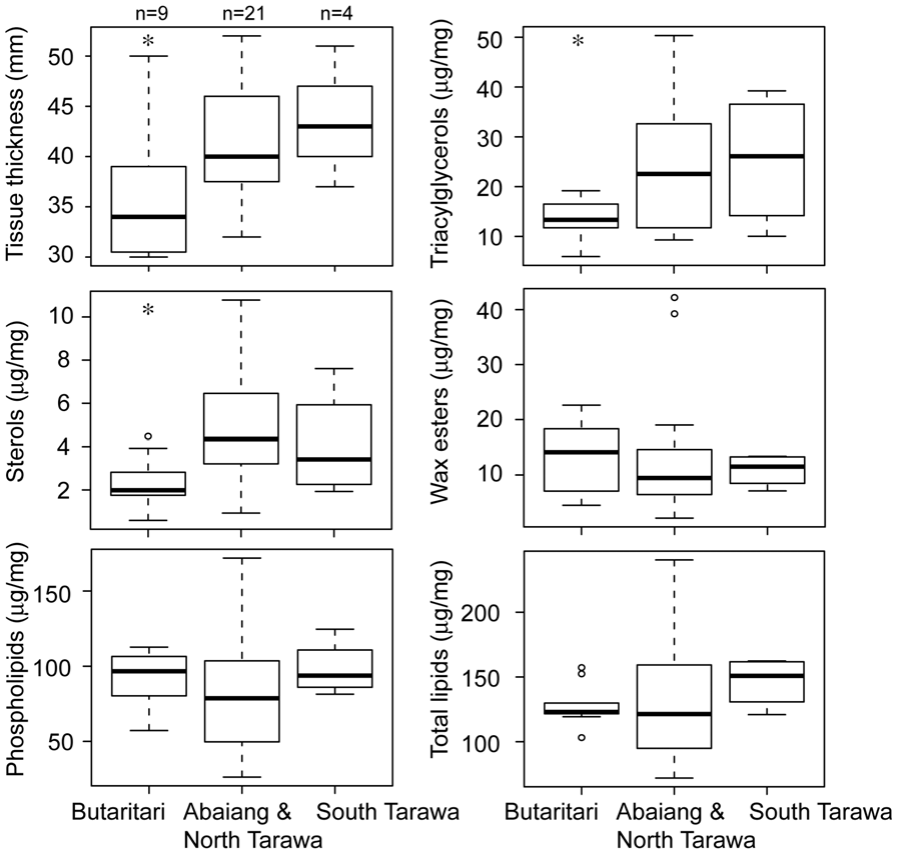
Boxplots of tissue thickness and lipid class concentrations for each island. Lipid concentrations are reported as µg lipid per mg total tissue. Asterisks indicate significant differences between Butaritari and other islands.

Measures of coral health at the time of sample collection were also significantly different between island groups (Figure 5). Corals from Abaiang and North Tarawa had significantly thicker tissue (mean 4.1 mm) than Butaritari (mean 3.6 mm) (p = 0.04, t-test), as well as significantly higher triacylglycerol concentrations (mean 23.4 versus 13.3 µg) (p = 0.03, permutation test) and sterol concentrations (mean 4.7 versus 2.4 µg) (p<0.01, t-test). There were no significant differences in wax esters, free fatty acids, phospholipids or total lipid concentrations between Abaiang and North Tarawa versus Butaritari. There were also no significant differences in lipid measures or tissue thickness between South Tarawa versus Abaiang and North Tarawa.

## Discussion

Butaritari experiences lower year-to-year variability in SST than Abaiang and Tarawa, which are more directly influenced by current and wind reversals during ENSO events [16], [47]. Although heat stress in 2004 was lower at Butaritari than at Abaiang and North Tarawa, massive *Porites sp.* corals at Butaritari experienced more severe bleaching as indicated by skeletal extension rate reductions and the occurrence of partial mortality scars. This evidence of reduced resistance to bleaching in 2004 in corals at Butaritari supports previous short-term manipulative experiments and long-term observational studies which found that higher background temperature variability or previous exposure to heat stress conferred bleaching resistance [15], [33]–[37]. Within individual colonies, we found that corals pre-exposed to higher thermal variability were better able to resist bleaching during a natural high temperature stress event. Our findings are also consistent with a study that tracked individual colonies and found prior exposure to high light reduced bleaching during a subsequent heat stress event [32].

The skeletal extension rate and partial mortality scar data for all three atolls suggest that massive *Porites sp.* corals in the Gilbert Islands could be, in general, more resistant to bleaching than corals in much of the tropics. Even in Butaritari, the decrease in extension rates and fraction of corals with partial mortality scars in 2004 was low despite levels of heat stress (mean maximum DHW of 10.4°C·week) which have caused and are predicted to cause severe bleaching and coral mortality in other regions of the world [43], [48], [49]. Coral reefs in the Gilbert Islands and other regions of the central equatorial Pacific experience higher year-to-year SST variability than most coral reefs in the tropics due to temperature fluctuations caused by ENSO. For example, the variability metric based on the standard deviation of the maximum monthly SST for the sites in South Tarawa, Abaiang and North Tarawa, and Butaritari is 69, 50, and 29% greater, respectively, than the median value for the world's coral reefs [16]. As such, the standard bleaching thresholds (e.g. DHW >4°C·week) may not accurately describe the likelihood of bleaching in locations with high inter-annual temperature variability like the Gilbert Islands; a method using the past inter-annual temperature variability to estimate the bleaching threshold may be more appropriate in these locations [16]. The evidence that bleaching resistance depends on thermal history, from the coral cores and from coral community surveys [47], suggests that the climatic variability of the Gilbert Islands, particularly those atolls closest to the equator (Abaiang and Tarawa), may have caused the corals to acclimatize to the observed level of heat stress.

The mechanisms by which such acclimatization could occur include (1) differences in symbiont type, (2) increased production of photoprotective compounds, or (3) increased production of antioxidants and heat-shock proteins. Different symbiont types can confer differential thermal tolerance [22], [23], and the types of symbionts harbored may change due to heat stress exposure, leading to increased bleaching resistance during later events [31]. Photoprotective compounds such as fluorescent proteins may increase bleaching resistance [29]; therefore, pre-exposure to a variable thermal environment might lead to increased production of photoprotective compounds, helping the corals cope with heat stress [33]. One study found higher antioxidant enzymes and heat-shock proteins in coral samples pre-exposed to light stress, which subsequently were less likely to bleach during heat stress [50]. These molecules can help prevent damage to coral tissue during oxidative stress [2], and likely reduce the occurrence of bleaching in response to oxidative stress [50]. It is possible that one or a combination of these and/or as-yet-unidentified factors caused the observed differences in bleaching resistance in the Gilbert Islands corals studied here.

In 2009–10, another ENSO-driven heat stress event occurred in the Gilbert Islands, with heat stress peaking in December. We found no significant effects from this heat stress event in skeletal extension rates in our coral cores, indicating that it did not cause as severe a bleaching event as in 2004. Because there was no apparent reduction in skeletal growth rates due to the milder 2009 heat stress event in our cores, we instead utilized the content of energetic lipids in coral tissue from each island as a more subtle measure of coral health after this event.

We found that triacylglycerol was significantly reduced in corals at Butaritari sampled in May of 2010 as compared to corals at Abaiang and North Tarawa. Of the two primary lipid energy sources in corals, triacylglycerol is more easily metabolized than wax esters and thus is commonly exhausted more rapidly by marine organisms during times of stress, such as starvation [51]. In *Porites compressa* nubbins that were experimentally bleached using heat stress, a reduction in triacylglycerol concentrations was observed 0, 1.5 and 8 months after bleaching [40]. While no reductions in extension rates were observed in cores taken seven months after the start of the 2009 heat stress event, reduced triacylglycerol concentrations at Butaritari are consistent with the conclusions drawn from extension rate reductions in 2004 that suggest corals there have a reduced capacity to cope with heat stress relative to Abaiang and Tarawa.

In contrast to the triacylglycerol concentrations, there was no significant difference in wax esters between the island groups. One study found that the response of wax esters to heat stress was delayed after bleaching, as that lipid class was not depleted at 0, 1.5, or 4 months after bleaching but was depleted 8 months after the bleaching occurred [40]. This is consistent with the role of wax esters class lipids as a long-term (i.e., slowly metabolized) energy source [51]. Our samples were collected approximately 5 months after the peak heat stress, and this delayed response may explain the lack of difference between island groups studied here. However, more studies investigating changes in lipid class concentrations at different times after bleaching are needed.

Corals in South Tarawa experience similar year-to-year temperature variability to those at Abaiang and North Tarawa, but are subject to greater local human-induced stress due to the proximity to the population and administrative centre of Kiribati [47]. Previous studies have found that local stress reduces bleaching resistance with a given amount of heat stress [11], [18]. The corals in South Tarawa did experience a greater reduction (29%) in extension rates in 2004 than the corals in North Tarawa and Abaiang (22%). However, the difference was not statistically significant, and the corals in South Tarawa were exposed to higher heat stress in 2004 than those in North Tarawa and Abaiang. In addition, after the 2009 heat stress event, there were no significant differences between coral tissue and lipids measures between South Tarawa versus Abaiang and North Tarawa (Figure 5).

The seeming resistance of corals in South Tarawa to bleaching may be due to the interaction of the local stressors with the effects of thermal history on the physiology of the corals. The lack of significant differences in tissue thickness or lipids measures between South Tarawa versus Abaiang and North Tarawa, despite higher heat stress in 2009 at South Tarawa, suggests that the sampled corals may benefit energetically due to increased food availability from surface runoff, appearing healthier several months after the heat stress event. For instance, one study found that corals in turbid, inshore environments actually had higher lipid stores than corals of the same species further offshore, probably due to higher rates of particle feeding [52]. The coral community on the outer reef in South Tarawa is different from that at the other outer reef sites studied; South Tarawa is dominated by the weedy encrusting species *Porites rus*, while at the other sites the coral community is dominated by massive *Porites spp., Heliopora spp., Montipora spp.* and *Favia spp.* [47]. The cover of *P. rus* in South Tarawa expanded after the 2004 bleaching event [47]; by 2010, *P. rus* comprised 66% and 80% of the coral cover at 5 m depth and 10 m depth, respectively, at our site TRW3 (unpublished survey data collected by SD in May 2010). One possibility is that the remaining massive *Porites sp.* corals we sampled at South Tarawa are highly resistant remnant colonies, and more susceptible massive *Porites sp.* colonies have already died off. The results from South Tarawa underscore the need to compare corals from similar environments to isolate the effects of a certain stressor.

These results suggest that the background temperature regime may affect coral resilience to bleaching, although the relationship may also depend on direct human stress on the coastal environment. This finding could eventually be useful in refining methods of bleaching prediction [16] and developing methods for identifying coral reefs or habitats that are more resilient to future warming. For example, locations subject to high historical temperature variability are likely to be less affected by future heat stress, and may be considered priorities for protection. Further data collection in the Gilbert Islands and other regions subject to ENSO-driven variability in temperature could help identify the effect of frequent heat stress events on the susceptibility of different taxa and different coral communities to bleaching and bleaching-related mortality.

## Supporting Information

Figure S1 **Annual coral calcification rates.** Calcification rates for individual coral cores standardized such that the long-term average for each record is equal to 1 g/cm^2^/year.
(TIF)

Figure S2 **Annual average coral density.** Annual average skeletal density for individual coral cores standardized such that the long-term average for each record is equal to 1 g/cm^3^/year.
(TIF)

Table S1 Average coral extension rate (cm/year), density (g/cm^3^/year), and calcification rates (extension * density; g/cm^2^/year) from individual sites, and from island groups used in study.
(DOC)

## References

[1] Brown B (1997). Coral bleaching: causes and consequences.. Coral Reefs.

[2] Lesser M (1997). Oxidative stress causes coral bleaching during exposure to elevated temperatures.. Coral Reefs.

[3] Downs C, Fauth JE, Halas JC, Dustan P, Bemiss J (2002). Oxidative stress and seasonal coral bleaching.. Free Radic Biol Med.

[4] Goreau TJ, Hayes RL (1994). Coral bleaching and ocean “hot spots”.. Ambio.

[5] Fitt W, Brown BE, Warner ME, Dunne RP (2001). Coral bleaching: interpretation of thermal tolerance limits and thermal threshold in tropical corals.. Coral Reefs.

[6] Strong A, Barrientos CS, Duda C, Sapper J (1997). Improved satellite techniques for monitoring coral reef bleaching.. Proc 8th Intl Coral Reef Symp.

[7] Harvell C, Kim K, Burkholder JM, Colwell RR, Epstein PR (1999). Emerging marine diseases–Climate links and anthropogenic factors.. Science.

[8] Lough J (2000). 1997–98: Unprecedented thermal stress to coral reefs?. Geophys Res Lett.

[9] McWilliams J, Côté IM, Gill JA, Sutherland WJ, Watkinson AR (2005). Accelerating impacts of temperature-induced coral bleaching in the Caribbean.. Ecology.

[10] Halley R, Hudson JH, Aronson R (2007). Fidelity of annual growth in *Montastraea faveolata* and the recentness of coral bleaching in Florida.. Geological Approaches to Coral Reef Ecology.

[11] Carilli J, Norris RD, Black B, Walsh SW, McField M (2010). Century-scale records of coral growth rates indicate that local stressors reduce coral thermal tolerance threshold.. Global Change Biol.

[12] Carilli JE, Godfrey J, Norris RD, Sandin SA, Smith JE (2010). Periodic endolithic algal blooms in *Montastraea faveolata* corals may represent periods of low-level stress.. Bull Mar Sci.

[13] Hoegh-Guldberg O (1999). Climate change, coral bleaching, and the future of the world's coral reefs.. Mar Freshw Res.

[14] Donner SD, Skirving WJ, Little CM, Oppenheimer M, Hoegh-Guldberg O (2005). Global assessment of coral bleaching and required rates of adaptation under climate change.. Global Change Biol.

[15] Thompson DM, Van Woesik R (2009). Corals escape bleaching in regions that recently and historically experienced frequent thermal stress.. Proc R Soc B: Biol Sci.

[16] Donner SD (2011). An evaluation of the effect of recent temperature variability on the prediction of coral bleaching events.. Ecol Appl.

[17] Baskett ML, Gaines SD, Nisbet RM (2009). Symbiont diversity may help coral reefs survive moderate climate change.. Ecol Appl.

[18] Wooldridge S (2009). Water quality and coral bleaching thresholds: Formalising the linkage for the inshore reefs of the Great Barrier Reef, Australia.. Mar Poll Bull.

[19] Berkelmans R, Willis BL (1999). Seasonal and local spatial patterns in the upper thermal limits of corals on the inshore Central Great Barrier Reef.. Coral Reefs.

[20] Marshall PA, Baird AH (2000). Bleaching of corals on the Great Barrier Reef: differential susceptibilities among taxa.. Coral Reefs.

[21] Yee SH, Santavy DL, Barron MG (2008). Comparing environmental influences on coral bleaching across and within species using clustered binomial regression.. Ecol Model.

[22] Berkelmans R, van Oppen MJH (2006). The role of zooxanthellae in the thermal tolerance of corals: a ‘nugget of hope’ for coral reefs in an era of climate change.. Proc R Soc B: Biol Sci.

[23] Sampayo EM, Ridgway T, Bongaerts P, Hoegh-Guldberg O (2008). Bleaching susceptibility and mortality of corals are determined by fine-scale differences in symbiont type.. Proc Natl Acad Sci.

[24] Anthony KRN, Kline DI, Diaz-Pulido G, Dove S, Hoegh-Guldberg O (2008). Ocean acidification causes bleaching and productivity loss in coral reef builders.. Proc Natl Acad Sci.

[25] Anthony KRN, Connolly SR, Hoegh-Guldberg O (2007). Bleaching, energetics, and coral mortality risk: Effects of temperature, light, and sediment regime.. Limnol Oceanog.

[26] Buddemeier RW, Fautin DG (1993). Coral bleaching as an adaptive mechanism.. BioScience.

[27] Rowan R (2004). Coral bleaching: Thermal adaptation in reef coral symbionts.. Nature.

[28] Coles SL, Brown BE (2003). Coral bleaching – capacity for acclimatization and adaptation.. Adv Mar Biol.

[29] Salih A, Larkum A, Cox G, Kühl M, Hoegh-Guldberg O (2000). Fluorescent pigments in corals are photoprotective.. Nature.

[30] Loya Y, Sakai K, Yamazato K, Nakano Y, Sambali H (2001). Coral bleaching: the winners and the losers.. Ecol Lett.

[31] Jones AM, Berkelmans R, van Oppen MJH, Mieog JC, Sinclair W (2008). A community change in the algal endosymbionts of a scleractinian coral following a natural bleaching event: field evidence of acclimatization.. Proc R Soc B: Biol Sci.

[32] Brown N, Dunne R, Goodson M, Douglas A (2002a). Experience shapes the susceptibility of a reef coral to bleaching.. Coral Reefs.

[33] Middlebrook R, Hoegh-Guldberg O, Leggat W (2008). The effect of thermal history on the susceptibility of reef-building corals to thermal stress.. J Exp Biol.

[34] Castillo K, Helmuth BST (2005). Influence of thermal history on the response of *Montastrea annularis* to short-term temperature exposure.. Mar Biol.

[35] Oliver TA, Palumbi SR (2011). Do fluctuating temperature environments elevate coral thermal tolerance?. Coral Reefs.

[36] Williams G, Knapp IS, Maragos JE, Davy SK (2010). Modeling patterns of coral bleaching at a remote central Pacific atoll.. Mar Poll Bull.

[37] Maynard J, Anthony K, Marshall P, Masiri I (2008). Major bleaching events can lead to increased thermal tolerance in corals.. Mar Biol.

[38] Leder J, Szmant AM, Swart PK (1991). The effect of prolonged “bleaching” on skeletal banding and stable isotopic composition in *Montastrea annularis*.. Coral Reefs.

[39] Suzuki A, Gagan MK, Fabricius K, Isdale PJ, Yukino I (2003). Skeletal isotope microprofiles of growth perturbations in *Porites* corals during the 1997–1998 mass bleaching event.. Coral Reefs.

[40] Rodrigues LJ, Grottoli AG, Pease TK (2008). Lipid class composition of bleached and recovering *Porites compressa* Dana, 1846 and *Montipora capitata* Dana, 1846 corals from Hawaii.. J Exp Mar Biol Ecol.

[41] Cantin NE, Cohen AL, Karnauskas KB, Tarrant AM, McCorkle DC (2010). Ocean warming slows coral growth in the central Red Sea.. Science.

[42] Lough J, Barnes DJ (2000). Environmental controls on growth of the massive coral *Porites*.. Jour Exp Mar Biol Ecol.

[43] Carilli J, Norris RD, Black BA, Walsh SM, McField M (2009). Local stressors reduce coral resilience to bleaching.. PLoS ONE.

[44] Barnes D, Lough JM (1992). Systematic variations in the depth of skeleton occupied by coral tissue in massive colonies of *Porites* from the Great Barrier Reef.. Jour Exp Mar Biol Ecol.

[45] Rodrigues L (2005). Physiology and biogeochemistry of bleached and recovering corals from Hawaii.

[46] Donner SD (2009). Coping with commitment: projected thermal stress on coral reefs under different future scenarios.. PLoS ONE.

[47] Donner SD, Kirata T, Vieux C (2010). Recovery from the 2004 coral bleaching event in the Gilbert Islands, Kiribati.. Atoll Res Bull.

[48] Strong A, Liu G, Meyer J, Hendee JC, Sasko D (2004). Coral Reef Watch 2002.. Bull Mar Sci.

[49] Eakin C, Morgan JA, Heron SF, Smith TB, Liu G (2010). Caribbean corals in crisis: Record thermal stress, bleaching, and mortality in 2005.. PLoS One.

[50] Brown BE, Downs CA, Dunne RP, Gibb SW (2002b). Exploring the basis of thermotolerance in the reef coral *Goniastrea aspera*.. Mar Ecol Prog Ser.

[51] Benson AA, Lee RF, Nevenzel JC (1972). Wax esters: Major marine metabolic energy sources.. Proc Biochem Soc.

[52] Anthony KRN (2006). Enhanced energy status of corals on coastal, high-turbidity reefs.. Mar Ecol Prog Ser.

