# Supplementary figures and images for: Historical Temperature Variability Affects Coral Response to Heat Stress
Source: PLoS One. 2012 Mar 30;7(3):e34418. doi: 10.1371/journal.pone.0034418 (PMC3316685; doi:10.1371/journal.pone.0034418)

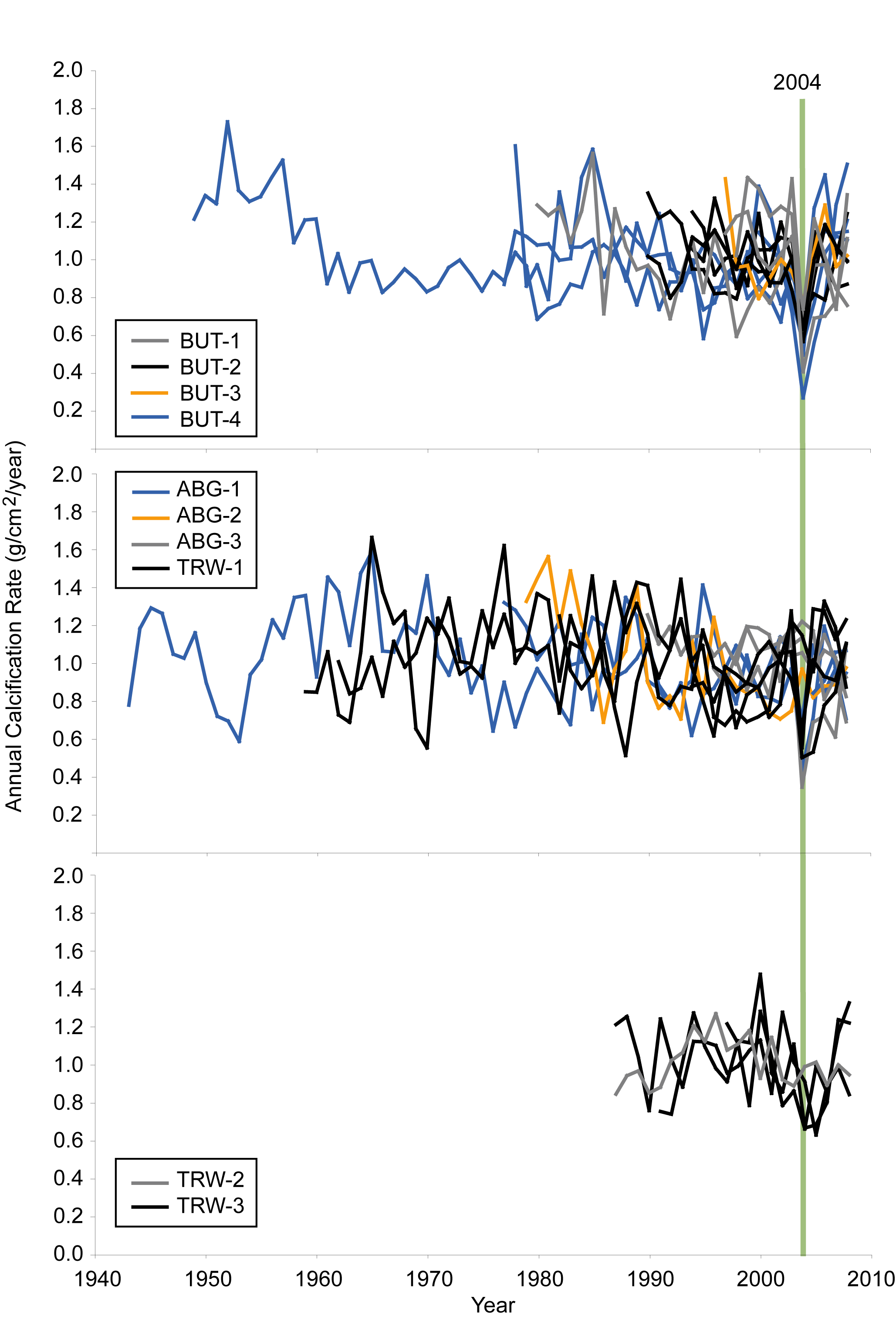

Supplement: Figure S1 — Annual coral calcification rates. Calcification rates for individual coral cores standardized such that the long-term average for each record is equal to 1 g/cm2/year. (TIF) [file pone.0034418.s001.tif]

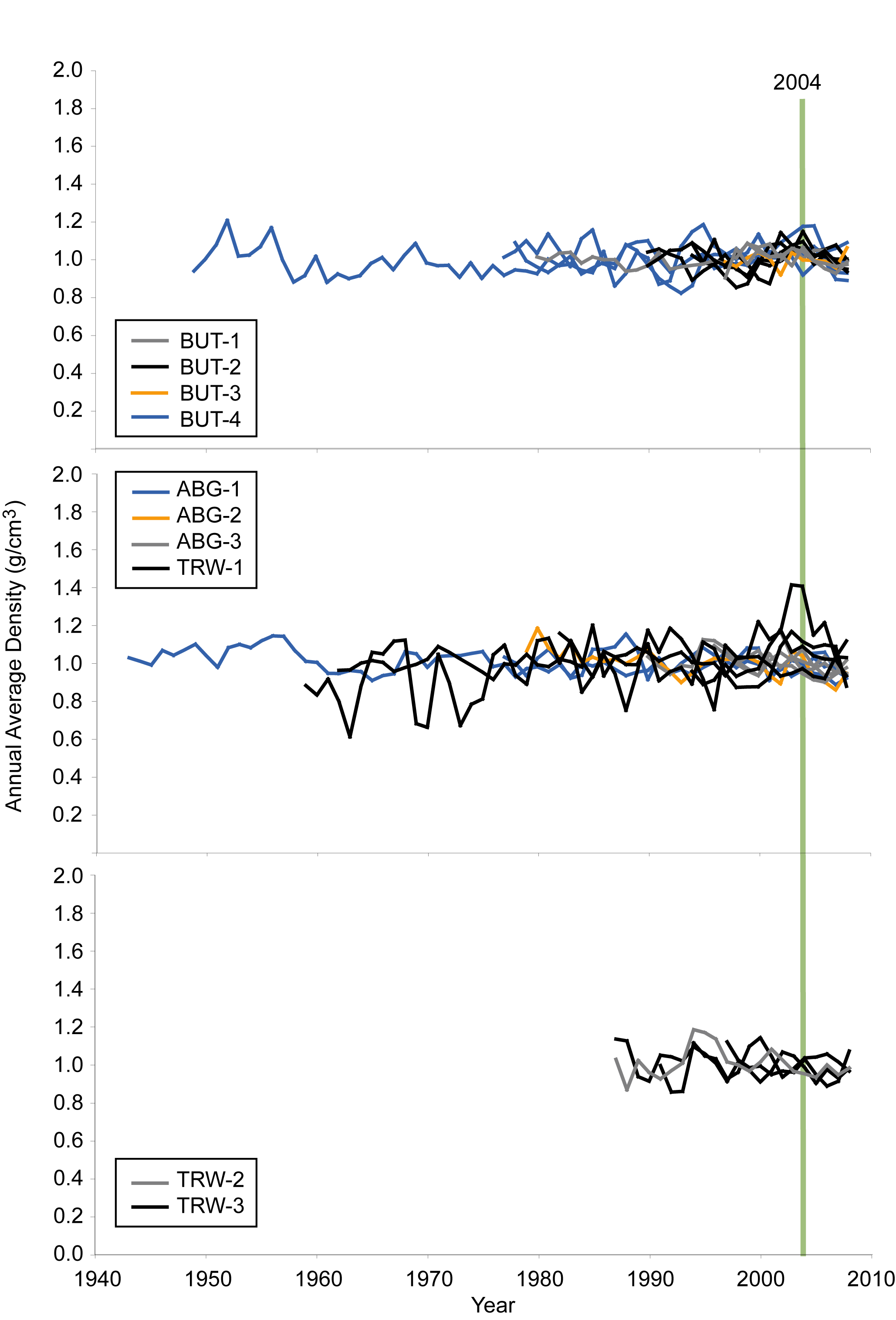

Supplement: Figure S2 — Annual average coral density. Annual average skeletal density for individual coral cores standardized such that the long-term average for each record is equal to 1 g/cm3/year. (TIF) [file pone.0034418.s002.tif]
