# Supplementary material for: Historical Temperature Variability Affects Coral Response to Heat Stress
Source: PLoS One. 2012 Mar 30;7(3):e34418. doi: 10.1371/journal.pone.0034418 (PMC3316685; doi:10.1371/journal.pone.0034418)
Supplement: Table S1 — Average coral extension rate (cm/year), density (g/cm3/year), and calcification rates (extension * density; g/cm2/year) from individual sites, and from island groups used in study. (DOC) [file pone.0034418.s003.doc]

**Table S1.** Average coral extension rate (cm/year), density (g/cm^3^/year), and calcification rates (extension * density; g/cm^2^/year) from individual sites, and from island groups used in study.

| **Site** | **Number of Cores** | **Average Extension Rate** | **Average Annual Density** | **Average Calcification Rate** |
| --- | --- | --- | --- | --- |
| BUT-1 | 3 | 1.15 | 1.59 | 1.84 |
| BUT-2 | 4 | 1.33 | 1.53 | 2.01 |
| BUT-3 | 1 | 0.76 | 1.69 | 1.27 |
| BUT-4 | 4 | 1.11 | 1.58 | 1.76 |
| ABG-1 | 3 | 1.04 | 1.51 | 1.57 |
| ABG-2 | 1 | 0.97 | 1.48 | 1.44 |
| ABG-3 | 4 | 1.32 | 1.45 | 1.90 |
| TRW-1 | 4 | 0.97 | 1.27 | 1.19 |
| TRW-2 | 1 | 0.60 | 1.45 | 0.86 |
| TRW-3 | 3 | 0.96 | 1.47 | 1.40 |
| Butaritari | 12 | 1.16 | 1.57 | 1.81 |
| Abaiang & N Tarawa | 12 | 1.10 | 1.41 | 1.54 |
| S Tarawa | 4 | 0.87 | 1.47 | 1.26 |
